# Gastrointestinal Development and Microbiota Responses of Geese to Honeycomb Flavonoids Supplementation

**DOI:** 10.3389/fvets.2021.739237

**Published:** 2021-10-18

**Authors:** Chenxin He, Huadong Wu, Yaning Lv, Hongnan You, Liqing Zha, Qin Li, Yani Huang, Jinghong Tian, Qiuchun Chen, Yiwen Shen, Shiyuan Xiong, Fuguang Xue

**Affiliations:** ^1^Nanchang Key Laboratory of Animal Health and Safety Production, Jiangxi Agricultural University, Nanchang, China; ^2^School of Foreign Language, Jiangxi Agricultural University, Nanchang, China; ^3^Jiangxi Province Key Laboratory of Animal Nutrition/Engineering Research Center of Feed Development, Jiangxi Agricultural University, Nanchang, China

**Keywords:** geese, honeycomb flavonoids, growth performance, gut microbiota, intestinal epithelium

## Abstract

**Background:** Geese are conventionally considered to be herbivorous, which could also be raised with concentrate feeding diets without green grass because of the similar gastrointestinal tract with other poultry. However, the geese gut microbiota profiles and their interactions with epithelial cells are still of limited study. Flavonoids were well-documented to shape gut microbiota and promote epithelial barrier functions individually or cooperatively with other metabolites. Therefore, in the present study, honeycomb flavonoids (HF) were supplemented to investigate the effects on growth performances, intestinal development, and gut microbiome of geese.

**Material and Methods:** A total of 400 1-day-old male lion-head geese with similar birth weight (82.6 ± 1.4 g) were randomly divided into five treatments: the control treatment (CON) and the HF supplementation treatments, HF was supplemented arithmetically to increase from 0.25 to 1%. Growth performance, carcass performances, and intestines' development parameters were measured to determine the optimum supplement. Junction proteins including ZO-1 and ZO-2 and cecal microbiota were investigated to demonstrate the regulatory effects of HF on both microbiota and intestinal epithelium.

**Results:** Results showed that 0.5% of HF supplement had superior growth performance, carcass performance, and the total parameters of gastrointestinal development to other treatments. Further research showed that tight junction proteins including ZO-1 and ZO-2 significantly up-regulated, while *Firmicutes* and some probiotics includin*g Clostridiales, Streptococcus, Lachnoclostridium*, and *Bifidobacterium*, remarkably proliferated after HF supplement. In conclusion, HF supplement in concentrate-diet feeding geese effectively increased the growth performances by regulating the gut microbiota to increase the probiotic abundance to promote the nutrient digestibility and fortify the epithelial development and barrier functions to facilitate the nutrient absorption and utilization.

## Introduction

In the latest years, the chicken gut metagenome and its modulatory effects on production performance, the heritability of chicken gut microbe, and the interactive regulation on feed efficiency between host genetics and gut microbiome have been well-studied (1, 2). However, little information could be acquired about geese. Geese are traditionally considered as herbivorous waterfowl which have been recently shown to be raised with concentrate feeding and without green grass (3, 4). Indeed, feeding with more concentrate significantly increased the growth and feed efficiency. Simultaneously, gastrointestinal morphology and gut microbiota altered with the change of diet. Therefore, to investigate the gastrointestinal development and gut microbiota, followed by the underlying interactive effects between the host and gut microbiota of geese, is of critical importance to geese production.

The gut microbiome is considered the host second genome, which emerged as a key determinant of many aspects including the capability of shaping developmental, physiological and reproductive phenotypes (5), the enhancement of feed digestibility and nutrient absorption throughout the digestive tract (2), and the inhibition on intestinal pathogenic bacteria (6). Further, unlike other environmental factors, the microbiomes may be transmitted between generations carrying the potentiality to alter traits beyond the limits of the host's genetics (7), and further worked on shaping the phenotypes of the progenies. Previous studies showed that gut microbiome is highly malleable by environmental factors (5) while symbiotic bacteria can increase bioavailable nutrient pools (7), which ultimately benefited the growth of the eukaryotic host.

Metabolic activity of the gut microbiota, especially the microbial secondary metabolites, expressed an essential role in maintaining homoeostasis and promoting growth of the host (8). Among which, flavonoids were extensively studied in shaping gut microbiota and epithelial barrier, and further applied in poultry production as a growth promoter and microbial regulator (9). Flavonoids and flavonoid metabolites have been well-documented to shape gut microbiota by inhibiting the growth of various pathogens and increasing beneficial genera such as *Bifidobacterium* and *Lactobacillus* because of their splendid anti-oxidation and free radical scavenging capabilities (10–12). Also, flavonoids are metabolized by the gut microbiota and intestinal tissues, resulting in modulation of intestinal cytokines and enhancement of gut epithelial barrier function (13). Moreover, flavonoids in the gastrointestinal tract were proven to modulate the activity of enzymes involved in lipids and carbohydrates, which further provided energy and substances for gut morphogenesis and functional maturation (14). All these benefits could in turn improve gut health by reducing the endotoxin production, increasing the conversion of primary into secondary bile acids, maintaining gut immune homeostasis, and promoting nutrients absorption (14).

Conventionally, flavonoids are extracted from plants, especially in Chinese medicine. Whereas, the honeycomb, which provides a residential place for bees and also is easy to acquire (15), was also virtually confirmed to contain a rich content of flavonoids (16, 17). Therefore, in the present study, honeycomb flavonoids (HF) were supplemented to determine the effects on growth performances, intestinal development, and gut microbiome of geese. We hypothesized that HF could increase the gut beneficial genera, modulate the epithelial development, and finally promote nutrient absorption and growth performance.

## Materials and Methods

Animal care and procedures followed The Chinese Guidelines for Animal Welfare, which was approved by the Animal Care and Use Committee of Jiangxi Agricultural University, with the approval number JXAULL-20201009.

### Experimental Design and Birds Feeding Procedure

A total of 400 one-day-old male lion-head geese with similar birth weight (82.6 g ± 1.4 g) was randomly divided into five treatments: the control treatment (CON) and the HF supplementation treatments, HF was supplementally arithmetically increased, which includes 0.25, 0.50, 0.75, and 1.0%. Each treatment contains four replications, with 20 birds in each replicate. All birds received the floor-rearing system, each replicate was allotted an 8-m long and 4-m wide compartment. Each compartment was 1 m high from the ground. All birds were provided with a two-phase feeding procedure (Day 0–21 as the starting phase, while Day 22–70 was the finishing phase), feed and water were provided *ad libitum* throughout the experiment. The room temperature was maintained at 37°C for the first week and then reduced by 3°C each week until reaching 24°C. The lighting schedule was 23 h light and 1 h dark during all experiment periods.

Raw honeycomb used in this study was provided by the Institute of Bee Research, Jiangxi Agricultural University, Jiangxi, China. Honeycomb was frozen at −18°C for 24 h, and then quickly pulverized and sieved through a 20-mesh screen for further investigation. HF in the present study was extracted in the laboratory using a 30-min long ultrasonic extracting procedure combined with a centrifugation at 3,000 rpm for 10 min. The liquid level of ultrasonic cleaner (Elmasonic X-tra Flexl, Elma, Konstanz, Germany) was kept between 3 and 5 cm. The post-extracting liquor was filtered after cooling and collected into an Erlenmeyer flask. The flavonoids content was measured for about 45% active ingredient.

For the experimental diets in each phase, a master-batch of the basal diet (negative control) was prepared in mash, and the additives were added afterward. The composition of the experimental diets and the nutrients are shown in Table 1.

**Table 1:** Composition of the experimental diets for geese.

**Ingredient**	**Starting phase**	**Finishing phase**
Corn	59.7	60.4
SBM, CP 43%	20.27	15.75
Alfalfa meal	11.22	13.65
Inferior powder	10	12
DL-Met	0.24	0.25
Stone powder	0.35	0.33
Calcium hydrophosphate DCP	2.53	2
Salt	0.35	0.32
Primix^a^	1	1
Total	100	100
**Level of nutrients (calculated)**		
ME/(kcal/kg)	2,810	2,790
CP	16.75	15.5
Ca	0.91	0.88
P	0.75	0.65
dLys	1.15	1.1
dMet	0.5	0.48
dCys	0.29	0.28
CF	5.5	6
dM+C	0.86	0.82

a *Primix: VA 12,000 IU/kg; VD_3_ 3,000 IU/kg; VE 7.5 IU/kg; VK_3_ 1.50 mg/kg; VB_1_ 0.6 mg/kg; VB_2_ 4.8 mg/kg; VB_6_ 1.8 mg/kg; VB_12_ 10 mg/kg; folic acid 0.15 mg/kg; niacinamide 30 mg/kg; pantothenic acid 10.5 mg/kg; Fe 80 mg, Cu 8 mg, Mn 80 mg, Zn 60 mg, Se 0.15 mg, and I 0.35 mg. CP, crude protein; CF, crude fiber*.

### Parameters Measurement

#### Growth Performance

The weight and feed consumption were weighed by replicate basis at hatching day, Day 21, and Day 70 to assess body weight gain (BWG), feed intake (FI), and feed conversion ratio (FCR). Geese were inspected thoroughly each day to record and remove any dead birds, and the feed intake was adjusted.

#### Carcass Performances

On Day 70, 2 birds per replication (total 40 samples) were randomly selected for the carcass characteristics measurement after 12-h fasting and sacrificed using electrical stunning. Eviscerated yield was calculated as the percentages of body weight (BW). Breast and thigh muscle yields were weighed and calculated as the percentages of eviscerated weight (EW). Abdominal fat percentage was calculated by abdominal fat weight/(abdominal fat weight + EW).

### Gastrointestinal Development

On Day 70, all parts throughout the gastrointestinal tract were sampled which includes muscular and glandular stomachs, duodenum, ileum, jejunum, cecum, and colorectum. The duodenum is connected to the muscular stomach, following with the jejunum and the ileum, which are in the second segment of the whole intestine. The cecum is located at the junction of the small intestine and the large intestine, which possessed two branched parallel bowel segments. Whereafter, the weight and length of each part of the intestine were measured to investigate the gastrointestinal development. Furthermore, tight junctions related genes ZO1 and ZO2 of intestinal epithelial cells which play important roles in maintaining the intestine barrier function also determined the expression quantity in both ileum and jejunum through reverse transcription-PCR (RT-PCR).

### Cecal Content Sampling and Microbiota Analysis

Cecal samples were collected from all slaughtered birds. All samples were rapidly frozen with liquid nitrogen and then stored at −80°C for further analysis. Based on the above-mentioned parameters, optimum supplement treatment was selected for the further microbiome analysis compared with the CON treatment. DNA from each sample was extracted using CTAB/SDS method followed by the measurement of DNA concentration and purity. The V4 region of the 16S rRNA gene was amplified using the universal primers 520F and 802R (F: GTGCCAGCMGCCGCGGTAA and R: GGACTACHVGGGTWTCTAAT). All PCR reactions were carried out with Phusion High-Fidelity PCR Master Mix (New England Biolabs). Samples with a bright main strip between 400 and 450 bp were chosen for further analysis. The mixture of PCR products was purified with Qiagen Gel Extraction Kit (Qiagen, Hilden, Germany) and, subsequently, sequencing libraries were generated using TruSeq^®^ DNA PCR-Free Sample Preparation Kit (Illumina, San Diego, CA). The library quality was assessed on the Qubit@ 2.0 Fluorometer (Thermo Scientific) and Agilent Bioanalyzer 2100 system. At last, the library was sequenced on Illumina HiSeq 4000 platform (Illumina Inc., San Diego, CA).

Quality filtering of raw tags was performed under specific filtering conditions to obtain the high-quality clean tags according to the Quantitative Insights Into Microbial Ecology (QIIME, V1.7.0) quality controlling process. Sequences within similarity >97% were assigned into the same operational taxonomic unit (OTU). For each representative sequence, the GreenGene Database was used based on the SILVA classifier algorithm to annotate taxonomic information.

### Statistical Analysis

Differential analysis of growth performances, carcass performances, and gastrointestinal development parameters was verified through a normal distribution test using SAS procedure “proc univariate data = test normal.” Subsequently, one-way ANOVA S-N-K test was applied to investigate the differences among the 5 treatments. Results were presented as mean ± SEM. OTU abundances of cecal bacteria first conducted a transformation of normal distribution using log_2_, and then Student's *T*-test of SAS 9.2 was applied for the differential analysis. Alpha diversity and beta diversity in our samples were calculated with QIIME (Version 1.7.0) and displayed with R software (Version 3.15.3). PCoA analysis was displayed by WGCNA package, stat packages, and ggplot2 package in R software (Version 3.15.3). The OTU abundance of ruminal bacteria was first transformed into normal distribution using the log_2_ transformation, and then the Student's *T*-test of SAS 9.2 was applied to analyze the differences of bacteria. *P* < 0.05 was significant. Spearman correlations between bacteria communities and production performances, carcass performances, and intestinal development parameters were assessed using the PROC CORR procedure of SAS 9.2 and then the correlation matrix was created and visualized in a heatmap format using R software (Version 3.15.3).

## Results

### Growth and Carcass Performances

Mortality and culling rate of each treatment was recorded daily, all treatments showed 1–2 deaths of birds throughout the trials, which indicated no significant changes were found among all treatments. Differential analysis of gradient HF supplement on growth performances was first evaluated including FI, BWG, and FCR in the starting phase, the finishing phase, and the whole phase. Results are shown in Table 2. No significant enhancive effects were acquired after HF supplement on both FI and BWG for either the starting or finishing phases. It is noteworthy that FCR showed a gradually decreasing trend with the increase of HF supplement in the finishing and the whole phase.

**Table 2:** Effects of honeycomb flavonoids on growth performances (*n* = 4).

**Items**	**CON**	**0.25%**	**0.50%**	**0.75%**	**1.00%**	***P*** **-value**	
Starting	BWG (g)	1,089 ± 36.4	1,102 ± 27.2	1,110 ± 25.6	1,107 ± 28.6	1,090 ± 31.6	0.123
Phase	FI (g)	3,613 ± 124.6	3,537 ± 137.8	3,529 ± 144.5	3,608 ± 128.9	3,535 ± 141.8	0.939
	FCR	3.32 ± 0.34	3.21 ± 0.29	3.18 ± 0.26	3.26 ± 0.31	3.24 ± 0.41	0.221
Finishing	BWG (g)	4,774 ± 59.8	4,784 ± 61.3	4,804 ± 56.7	4,795 ± 64.2	4,789 ± 67.6	0.797
Phase	FI (g)	17,368 ± 267.7	17,473 ± 284.3	17,467 ± 277.6	17,270 ± 291.4	17,161 ± 299.8	0.136
	FCR	3.64 ± 0.29	3.65 ± 0.31	3.64 ± 0.28	3.60 ± 0.37	3.58 ± 0.32	0.292
Whole	BWG (g)	5,863 ± 95.2	5,886 ± 88.6	5,914 ± 82.3	5,902 ± 92.4	5,879 ± 98.2	0.116
Phase	FI (g)	20,981 ± 391.6	21,010 ± 411.7	20,996 ± 413.5	20,879 ± 419.8	20,696 ± 431.6	0.224
	FCR	3.58 ± 0.38	3.57 ± 0.30	3.55 ± 0.27	3.54 ± 0.34	3.52 ± 0.36	0.437

*CON, control diet; SEM, standard error of the mean*.

Subsequently, carcass characteristics were measured, and the results are presented in Table 3. Totally, HF supplement significantly promoted the dressed weight and semi-eviscerated weight while it tended to promote the eviscerated weight. Besides, supplemented with 0.5 and 0.75% of HF significantly increased the tight weight compared with other treatments. No significant changes were found for other parameters after HF supplement.

**Table 3:** Effects of honeycomb flavonoids supplement on carcass performances (n = 8).

**Items**	**CON**	**0.25%**	**0.50%**	**0.75%**	**1.00%**	***P*** **-value**
Body weight (g)	5,945 ± 95.6	5,968 ± 88.9	5,996 ± 82.5	5,984 ± 92.6	5,961 ± 98.4	0.123
Dressed weight (g)	5,258 ± 91.3^b^	5,318 ± 87.6^a^	5,320 ± 81.6^a^	5,326 ± 91.7^a^	5,317 ± 97.3^a^	0.034
Semi-eviscerated weight (g)	4,741 ± 85.8^b^	4,772 ± 83.7^ab^	4,806 ± 78.9^a^	4,822 ± 87.6^a^	4,813 ± 91.4^a^	0.047
Eviscerated weight (g)	4,267 ± 87.8	4,271 ± 83.1	4,334 ± 79.8	4,349 ± 82.7	4,350 ± 86.4	0.068
Abdominal fat (g)	132.7 ± 11.4	131.2 ± 12.6	129.8 ± 10.9	126 ± 11.3	123.8 ± 10.8	0.343
Breast (g)	442.9 ± 26.8	464.9 ± 27.9	468.3 ± 25.3	437.2 ± 31.3	482.6 ± 33.8	0.064
Thigh (g)	657.7 ± 15.7^b^	654.5 ± 13.8^b^	670.0 ± 11.3^a^	679.2 ± 15.6^a^	660.6 ± 13.3^ab^	0.038
Dressing percentage (%)	88.45 ± 0.52	89.12 ± 0.59	88.72 ± 0.68	89.01 ± 0.81	89.2 ± 0.84	0.621
Semi-eviscerating percentage (%)	79.74 ± 0.87	79.96 ± 0.92	80.15 ± 0.88	80.58 ± 1.01	80.74 ± 0.93	0.082
Eviscerating percentage (%)	71.78 ± 0.96	71.57 ± 0.88	72.27 ± 0.94	72.67 ± 1.03	72.98 ± 1.13	0.217
Abdominal percentage (%)	3.11 ± 0.29	3.07 ± 0.24	3.0 ± 0.27	2.9 ± 0.19	2.85 ± 0.31	0.483
Breast percentage (%)	10.68 ± 0.68	10.88 ± 0.71	10.81 ± 0.75	11.05 ± 0.81	11.09 ± 0.79	0.557
Tight percentage (%)	15.41 ± 0.48	15.32 ± 0.44	15.46 ± 0.38	15.62 ± 0.46	15.19 ± 0.49	0.37

a, b *Means within a row with different letters differed significantly (P < 0.05); SEM, standard error of the mean; CON, control diet*.

### Gastrointestinal Development and Cecal Microbiota

#### Gastrointestinal Development

Gastrointestinal development determined the nutrient digestibility and might be the determinant on FCR of the whole body. Therefore, in the present research, development of the whole digestive tract was measured which included muscular stomach, glandular stomach, duodenum, jejunum, ileum, cecum, and colorectum. All the results are shown in Table 4. Based on the results, gastro-development showed a significant inflexion with the increase of HF supplement and 0.5% of HF supplement performed superior to other treatments in the total parameters of gastrointestinal development. Notably, the jejunum, ileum, and cecum showed the best performance in 0.5% treatment. No other significant differences were detected among all the treatments. Therefore, supplement with 0.5% of HF was chosen for the further cecal microbiota analysis based on the growth performances, carcass performances, and the intestinal development parameters.

**Table 4:** Effects of honeycomb flavonoids on gastrointestinal development (*n* = 8).

**Items**	**CON**	**0.25%**	**0.50%**	**0.75%**	**1.00%**	***P*** **-value**
Muscular stomach (g)	145.5 ± 9.6	138.6 ± 8.7	136.4 ± 9.4	136.1 ± 8.9	130.5 ± 10.1	0.364
Glandular stomach (g)	15.88 ± 2.61	14.04 ± 2.33	13.7 ± 2.25	16.86 ± 2.98	14.5 ± 2.38	0.152
Duodenum (g)	14.53 ± 0.97	15.33 ± 1.21	14.82 ± 0.89	15.21 ± 1.13	15.21 ± 1.16	0.231
Jejunum (g)	34.06 ± 2.03^b^	37.38 ± 1.99^a^	35.30 ± 1.78a^b^	33.31 ± 2.03^b^	34.31 ± 2.03^b^	0.048
Ileum (g)	29.95 ± 1.13^b^	29.55 ± 1.21^b^	31.45 ± 1.22^a^	28.26 ± 1.29^b^	29.89 ± 1.33^b^	0.041
Cecum (g)	8.76 ± 0.21^b^	9.03 ± 0.18^ab^	9.45 ± 0.27^a^	9.19 ± 0.29^ab^	8.87 ± 0.19^b^	0.037
Colorectum (g)	9.6 ± 1.08	8.69 ± 1.13	9.13 ± 1.09	9.31 ± 1.11	9.35 ± 1.21	0.076
Duodenum (cm)	50.22 ± 1.79	50.17 ± 2.01	50.5 ± 2.13	50.96 ± 1.97	50.65 ± 2.08	0.743
Jejunum (cm)	115.02 ± 3.56^a^	107.58 ± 4.29^b^	104.43 ± 3.97^b^	101.93 ± 2.74^c^	109.36 ± 2.64^b^	0.022
Ileum (cm)	99.42 ± 2.33^b^	101.37 ± 2.76^b^	103.75 ± 2.64^ab^	106.81 ± 1.97^a^	107.39 ± 2.18^a^	0.037
Cecum (cm)	57.61 ± 2.74	58.58 ± 3.12	60.98 ± 2.97	56.23 ± 3.12	60.18 ± 3.22	0.243
Colorectum (cm)	15.58 ± 0.97	15.78 ± 1.13	15.54 ± 1.22	15.87 ± 1.19	15.38 ± 1.34	0.328

a, b, c *Means within a row with different letters differed significantly (P < 0.05); SEM, standard error of the mean; CON, control diet*.

#### Expression of ZO1 and ZO2

**Figure 1:**
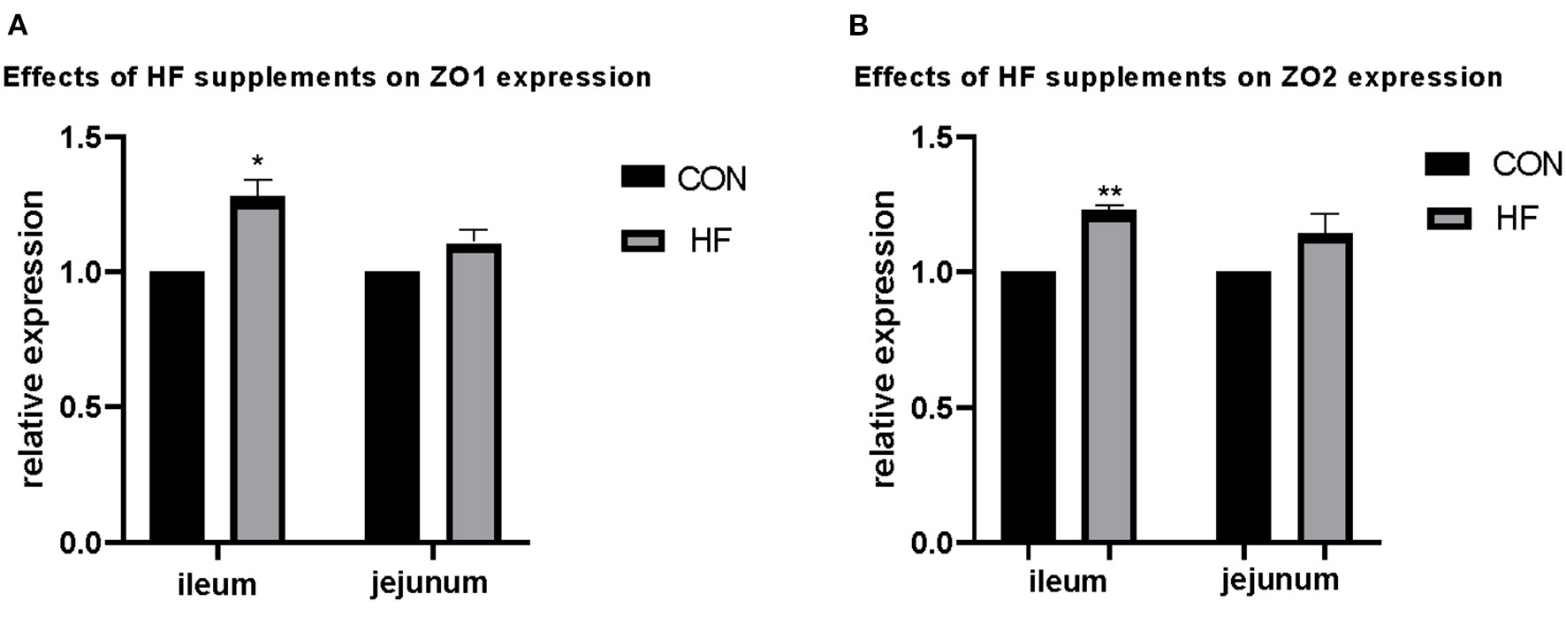
Effects of honeycomb flavonoids (0.5% supplementation) on the relative expression of ZO1 and ZO2 in the ileum and jejunum. **(A)** Effects of honeycomb flavonoids on the relative expression of ZO1 in the ileum and jejunum. **(B)** Effects of honeycomb flavonoids on the relative expression of ZO2 in the ileum and jejunum. *significant correlation; **very significant correlation.

Apart from the weight and length, the epithelial absorption and barrier functions account for the gross of gastrointestinal development. Structural integrity of gut epithelium represented a better function of the intestine. The tight junction proteins played an important role in the epithelial integrity and thus the genes with encodes tight junction proteins were determined to partially illustrate the effects of HF. Results are shown in Figure 1. The expression of ZO1 and ZO2 in both the ileum and the jejunum were detected. Expression of ZO1 and ZO2 was up-regulated after HF supplementation in both ileum and jejunum, and both genes were significantly up-regulated in the ileum compared with the control treatments. No significant changes were found in the jejunum.

### Cecal Microbiota

The 16S rRNA gene amplicon sequences from cecal contents of both CON and HF treatment samples were conducted to investigate the effects of HF supplementation treatments on gastrointestinal microbiota. Taxonomy results of all bacteria are shown in Supplementary Table 1. A total of 10 phyla and more than 200 genera were identified in the present study, the average length of sequence reads was about 410 nt. All the results were subsequently analyzed for α-diversity and β-diversity.

#### α-Diversity

α-diversity was applied in analyzing complexity of species diversity through Chao1, Shannon, Simpson, and ACE indexes, and all results are displayed in Table 5. Indexes including Chao1, Shannon, and ACE increased after HF supplement, while noticeably the Shannon index is significantly increased after HF treatment, which indicated that HF supplement increased the cecal bacterial diversity.

**Table 5:** Effects of honeycomb flavonoids (0.5% of HF) on α-diversity of cecal contents bacterial communities (*n* = 8).

**Items**	**CON**	**HF**	***P*** **-value**
Shannon	3.93 ± 0.061	4.06 ± 0.059	0.031
Simpson	0.07 ± 0.014	0.05 ± 0.017	0.126
ACE	495.2 ± 12.34	515.1 ± 15.64	0.364
Chao	498.8 ± 9.59	505.6 ± 11.23	0.447

*CON, control diet; HF, honeycomb flavonoids supplement treatment; SE, standard error*.

#### β-Diversity

**Figure 2:**
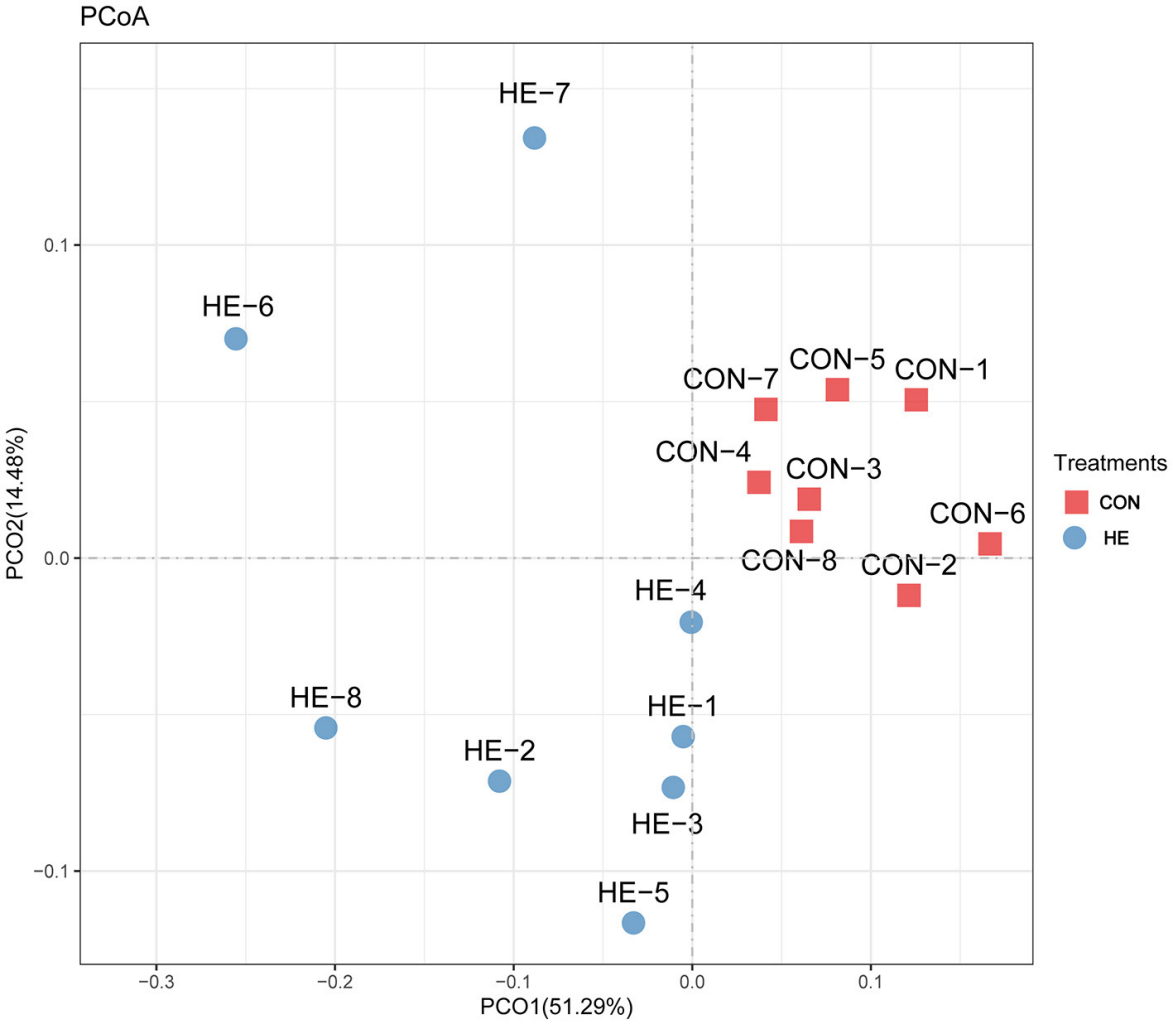
Principal coordinate analysis (PCoA) on community structures of the cecal microbiota after honeycomb flavonoids (0.5% supplementation) treatment. CON, control diet; HF, honeycomb flavonoids supplement treatment.

Principal coordinates analysis (PCoA), which mainly clarified the monolithic discrepancy of bacterial profiles between HF and CON treatments, was first processed. As shown in Figure 2, PCoA axes 1 and 2 account for 51.29 and 14.48%, respectively. Bacterial communities after HF treatment could be clearly separated from those in CON treatment through PCoA axes 1 and 2, which indicates a significant alteration of bacterial communities took place after HF supplements.

Whereafter, differential analysis on the abundances of different bacteria in both the phyla and the genera levels were investigated, and all results are shown in Tables 6, 7. *Firmicutes* located the dominant phylum, while *Bacteroidetes* and *Proteobacteria* accounted for the 2nd and 3rd of the total microbiota, respectively. HF significantly promoted the proliferation of *Firmicutes* and *Tenericutes Bacteroidetes* while suppressed *Bacteroidetes, Verrucomicrobia*, and *Fibrobacteres*. No significant changes were investigated for other phyla.

**Table 6:** Effects of honeycomb flavonoids (0.5% of HF) on the relative abundances of cecal bacterial communities in the level of phyla (*n* = 8).

**Phyla**	**CON (%)**	**HF (%)**	***P*** **-value**
*Bacteroidetes*	14.92 ± 2.36	11.07 ± 2.46	0.163
*Firmicutes*	78.73 ± 3.24	83.73 ± 2.19	0.042
*Proteobacteria*	2.60 ± 0.64	3.19 ± 0.58	0.569
*Actinobacteria*	0.61 ± 0.16	0.83 ± 0.21	0.052
*Spirochaetae*	0.78 ± 0.36	0.34 ± 0.29	0.192
*Tenericutes*	1.32 ± 0.27	0.47 ± 0.19	0.029
*Verrucomicrobia*	0.14 ± 0.07	0.04 ± 0.02	0.009
*Fibrobacteres*	0.10 ± 0.07	0.07 ± 0.04	0.392
*Others*	0.81 ± 0.47	0.26 ± 0.25	0.111

*CON, control diet; HF, honeycomb flavonoids supplement treatment*.

**Table 7:** Effects of honeycomb flavonoids (0.5% of HF) on the relative abundances of cecal bacterial communities in the level of genus (*n* = 8).

**Items**	**CON (%)**	**HF (%)**	***P*** **-value**
*g__Faecalibacterium*	16.00 ± 2.78	12.22 ± 3.11	0.091
*g__Prevotella*	10.77 ± 2.16	17.77 ± 2.54	0.005
*g__Ruminococcaceae*	12.21 ± 3.12	9.01 ± 2.01	0.301
*g__Alistipes*	9.98 ± 2.11	8.41 ± 1.79	0.395
*g__Ruminococcus*	9.38 ± 0.97	10.64 ± 1.47	0.216
*g__Lachnospiraceae*	7.51 ± 1.02	8.01 ± 1.21	0.771
*g__Prevotellaceae*	6.15 ± 0.96	5.86 ± 0.79	0.544
*g__Lactobacillus*	4.69 ± 0.34	5.82 ± 0.67	0.092
*g__Escherichia Shigella*	2.91 ± 0.14	1.29 ± 0.21	<0.001
*g__Anaerotruncus*	2.75 ± 0.64	2.16 ± 0.37	0.787
*g__Lachnoclostridium*	2.30 ± 0.21	3.30 ± 0.27	0.004
*g__Butyricicoccus*	2.39 ± 0.22	2.41 ± 0.16	0.273
*g__Eisenbergiella*	2.11 ± 0.31	1.96 ± 0.24	0.417
*g__Clostridiales*	1.17 ± 0.11	1.72 ± 0.16	0.001
*g__Sellimonas*	2.11 ± 0.18	1.12 ± 0.11	0.031
*g__Streptococcus*	0.63 ± 0.09	0.94 ± 0.11	0.041
*g__Enterococcus*	0.43 ± 0.07	0.57 ± 0.14	0.224
*g__Flavonifractor*	0.50 ± 0.11	0.55 ± 0.09	0.446
*g__Succinivibrio*	0.09 ± 0.01	0.13 ± 0.07	0.143
*g__Bifidobacterium*	0.02 ± 0.004	0.03 ± 0.003	0.039
Others	5.90 ± 0.24	6.07 ± 0.31	0.161

*CON, control diet; HF, honeycomb flavonoids supplement treatment*.

As referred to the genera level, *Faecalibacterium, Ruminococcaceae*, and *Ruminococcus* accounted for the top three genera of both CON and HF treatments. Compared with CON, HF supplement significantly increased the *Prevotella, Clostridiales, Streptococcus, Lachnoclostridium*, and *Bifidobacterium*, while significantly decreased the abundances of *Escherichia Shigella* and *Sellimonas. Faecalibacterium* showed a decreasing trend after HF treatment, and no significant changes of other genera were investigated.

### Interactive Analysis Between Cecal Microbiota and Production Performances, Carcass Performances, and Intestinal Development Parameters

**Figure 3:**
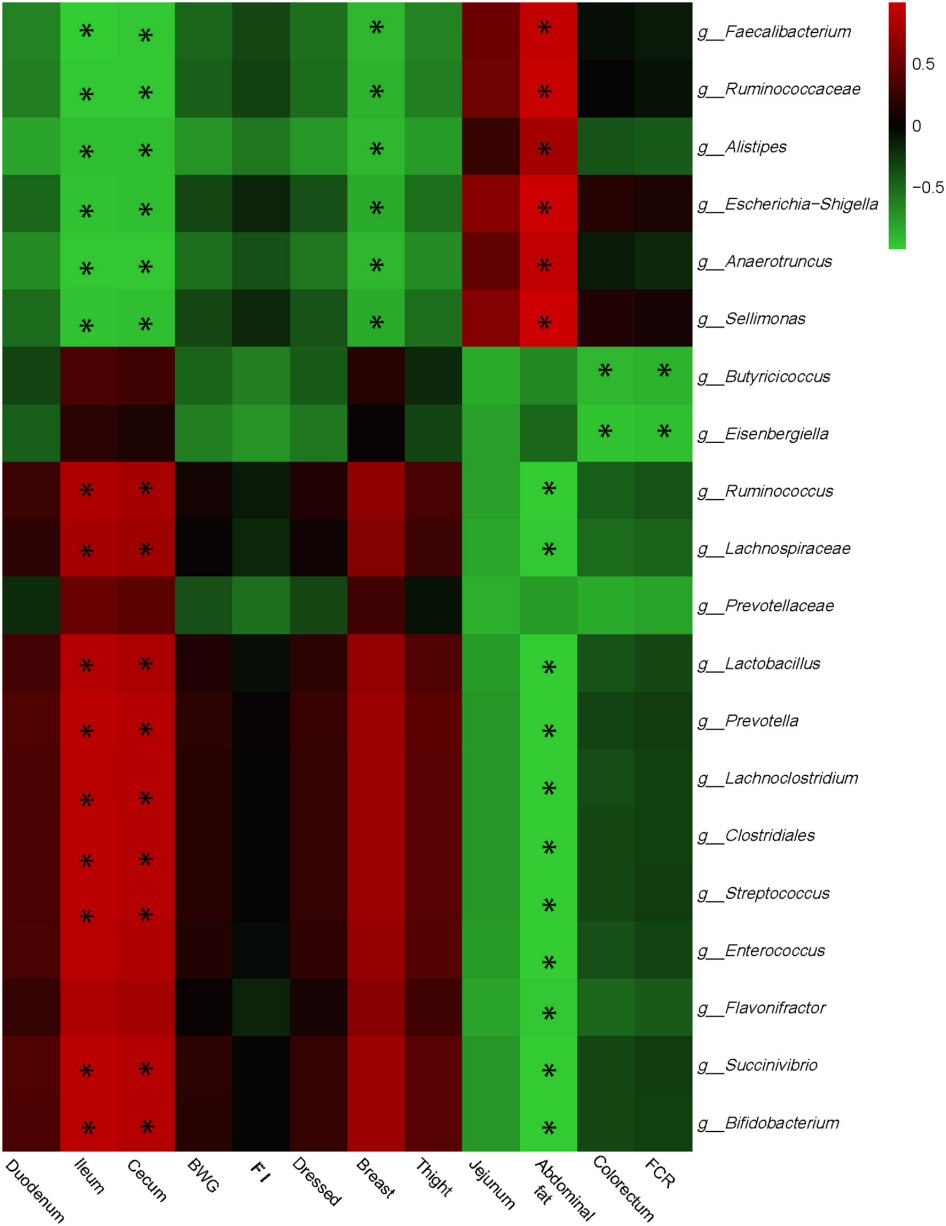
Correlation analyses between abundances of cecal bacteria and growth performances, carcass performances, and intestinal development parameters on the level of genera. The red color represents positive correlation while the green color represents a negative correlation. *Means a significant correlation (|*r*| > 0.55, *P* < 0.05).

Interactive analysis between the most abundant bacteria and production performance, carcass performance, and intestinal development parameters were conducted and the result is shown in Figure 3.

Integrally, phenotype parameters could be separated into two big clusters. The first, which mainly consists of ileum, cecum, FI, and breast, was positively correlated with *Butyricicoccus, Prevotella, Clostridiales, Streptococcus, Lachnoclostridium*, and *Bifidobacterium*, while negatively correlated with *Faecalibacterium, Ruminococcaceae, Escherichia Shigella*, and *Sellimonas*. The other cluster showed the reverse correlations with bacterial genera compared with the first one which included jejunum, abdominal fat, and FCR. Especially, *Butyricicoccus* and *Eisenbergiella* remarkably negatively correlated with FCR, which may provide the target for reducing FCR and promoting feed efficiency.

## Discussion

Geese are generally considered the herbivore poultry. However, unlike the ruminants who possess the high-digestibility rumen or the rabbits who possess the powerful cecum, the geese gastrointestinal tract is similar to other poultries. Therefore, how the intestinal development and the gut constituent shaped the herbivority of geese fascinated researchers. Previous studies indicated the geese could also be reared like chickens with high-concentrate diets (18), and the growth performances changed with different nutritional levels (19, 20). Whereas, the geese gut microbiota profiles and their interactions with epithelial cells are still of limited cognition. To our knowledge, this might be the first time that the development of every intestinal segment and the interactions between gut microbiome and the host of geese are investigated.

### Effects of Honeycomb Flavonoids on Intestinal Development

The intestinal functions typically regulated by the epithelial absorptivity and gut microbiota digestibility. The nutrients' absorption mainly occurs at the upper parts of the gastrointestinal tract, which is regulated by high levels of acids, abundant digestive enzymes, and antimicrobials (21). Besides, the intestinal epithelial cells (IECs) exist as a layer of columned cells that generated a functional barrier to protect the intestinal mucosa from pathogenic microorganisms, and an interchange channel to absorb nutrients into circulation (22). The epithelium also contains plentiful functional proteins, which conduct the substances interchange or achieve informative communication with other metabolites as the target spot (23).

Flavonoids in the intestines were considered as efficient antimicrobial metabolites, which showed a broad-spectrum antimicrobial capacity and inhibited most pathogenic micro-organisms and thus improved epithelium development. Moreover, flavonoids in the intestinal tract interacted with the functional proteins including inhibition of inflammatory signaling such as nuclear factor-kappa B (NK-kB) and up-regulation of tight-junction proteins such as ZO1 to fortify the intestinal tight junction barrier and structure (18, 24). In the present study, the expression of tight-junction proteins ZO1 and ZO2 showed a significant increase with HF supplementation. This might partly enhance intestinal barrier functions and further intestinal absorptivity.

### Effects of Honeycomb Flavonoids on Gut Microbiota

Apart from the enzymatic digestion in the upper parts of the gastrointestinal tract, colonic and cecal conditions support a diverse community of bacteria that are capable of fermenting complex substances undigested in the small intestine (21, 25). In our research, the microbiota community is dominated by bacteria, with more than 90% of the species belonging to *Firmicutes* and *Bacteroidetes*, which is in line with Tremaroli and Backhed (26). What is noteworthy is that the ratio of *Firmicutes* to *Bacteroidetes* was positively correlated with the energy metabolism, the higher *Firmicutes* boosts the concentrate's digestibility, and thus more energy was provided. In our research, *Firmicutes* significantly increased after HF supplement. This may partly explain the increased body weight after HF supplement.

Particularly, *Lactobacillus* and *Bifidobacterium*, which played important roles in maintaining intestinal homeostasis and fortifying the intestinal mucus layer (27, 28) in the present study were significantly proliferated in HF treatment. Higher abundance of cecal *Lactobacillus* positive regulated a better feed efficiency (2), while increased *Bifidobacterium* correlated with reduced adiposity and levels of microbe-derived inflammatory molecules (29). These changes protected intestinal structure, enhanced intestinal barrier functions, and further promoted the intestinal absorptivity.

### The Underlying Interactions Between Microbiota and Gut Epithelium to HF Supplement

To date, host and bacteria interactions turned into a great causal factor that shaped the gastrointestinal tract, affected host fitness, growth rates, and carrying capacity through providing more energy and scavenging inflammatory molecules (7, 30). Thus, the host-associated bacteria interacting with the epithelial barrier is thought to be the main underlying mechanism that impacts the geese growth performance. Just as we here focused on the nutrient's metabolism, the uppermost energy supplier such as carbohydrates, and the main nutritional anti-inflammatory metabolite—bile acid—are considered as the main substances mediating the connection between host and bacteria to the HF supplement (31, 32).

The carbohydrates, including cellulose, xylans, and starch, yield the most energy by colon and cecum microbiota. The further fermented products such as short-chain fatty acids (SCFAs) have profound effects on gut health and energy absorption and utilization (26). Of the SCFAs produced from microbial fermentation, butyrate is particularly important as an energy substrate for epithelial development and cellular metabolism in the colonic and cecal epithelium (33). In the present research, the main butyrate generating bacteria such as *Clostridium* and *Butyricicoccus* remarkably increased after HF supplement (34, 35). This might indicate that HF stimulated the proliferation of butyrate generating bacteria and further provided more energy for gut epithelial development.

Bile acid (BA) metabolism might be another causative factor that regulated intestinal absorptivity. In recent research, the BA signaling pathway was proved to be critical in both lipid and carbohydrate metabolism, also in maintaining esoteric glucose and cholesterol homeostasis as well as immune states (36, 37). In addition, bile acids can regulate gut microbial composition both directly and indirectly by activation of innate immune response genes in the small intestine (37). BA is also a crucial secondary metabolite that is utilized by gut microbiota and communicated with epithelial cells. Research further confirmed that over 80% of microbiota-host interactions were with secondary Bas (8). Intriguingly, flavonoids could be responsible for increased BA production (38). These findings provided us a strong promotive effect of HF on gastrointestinal development and growth performances.

## Conclusion

**Figure 4:**
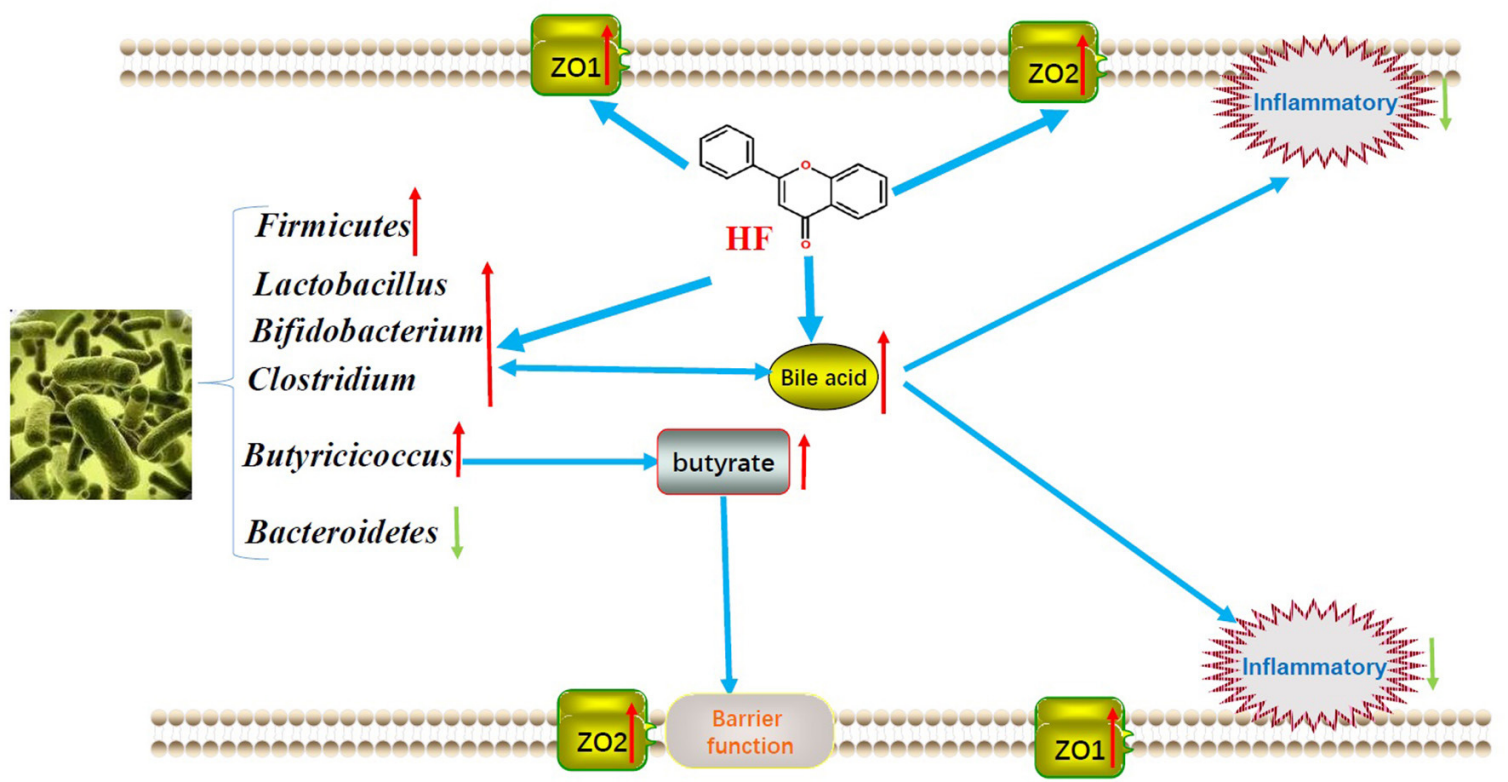
Underlying mechanism of honeycomb flavonoids on the intestinal absorption of geese.

In summary, HF supplement in concentrate-diet feeding geese may effectively increase the growth performances by regulating the gut microbiota to promote the nutrient digestibility and fortifying the epithelial development and barrier functions to facilitate the nutrient absorption and utilization. Finally, we showed the predicted mechanism model of HF on geese growth performance in Figure 4 to make it visualizable. The findings in this research might provide an efficacy in raising geese with concentrate diets.

## Data Availability Statement

The datasets presented in this study can be found in online repositories. The names of the repository/repositories and accession number(s) can be found in the article/Supplementary Material.

## Ethics Statement

The animal study was reviewed and approved by Animal Care and Procedures followed the Chinese Guidelines for Animal Welfare, which was approved by the Animal Care and Use Committee of Jiangxi Agricultural University, with the approval number JXAULL-20201009.

## Author Contributions

FX designed the study. CH, HW, and LZ conducted the experiment. FX and HY contributed to the manuscript writing and English editing. QL, YH, JT, QC, YS, and SX contributed to parameter measurement and the data analysis. All authors contributed to the article and approved the submitted version. All authors carefully read and are accountable for all aspects of the work.

## Conflict of Interest

The authors declare that the research was conducted in the absence of any commercial or financial relationships that could be construed as a potential conflict of interest.

## Publisher's Note

All claims expressed in this article are solely those of the authors and do not necessarily represent those of their affiliated organizations, or those of the publisher, the editors and the reviewers. Any product that may be evaluated in this article, or claim that may be made by its manufacturer, is not guaranteed or endorsed by the publisher.

## Supplementary Material

Supplementary Table 1 Taxonomy results of cecal microbiota in both control and HF supplement treatments.
